# Effect of characteristics on the clinical course at the initiation of treatment for human immunodeficiency virus infection using dimensionality reduction

**DOI:** 10.1038/s41598-023-31916-x

**Published:** 2023-04-04

**Authors:** Yunsu Choi, Bo Youl Choi, Sang Il Kim, Jungsoon Choi, Jieun Kim, Bo Young Park, Soo Min Kim, Shin-Woo Kim, Jun Yong Choi, Joon Young Song, Youn Jeong Kim, Hyo Youl Kim, Jin-Soo Lee, Jung Ho Kim, Yoon Hee Jun, Myungsun Lee, Jaehyun Seong

**Affiliations:** 1 Department of Preventive Medicine, College of Medicine, Hanyang University, Seoul, Republic of Korea; 2 Institute for Health and Society, College of Medicine, Hanyang University, Seoul, Republic of Korea; 3 Division of Infectious Disease, Department of Internal Medicine, Seoul St. Mary’s Hospital, College of Medicine, The Catholic University of Korea, Seoul, Republic of Korea; 4 Department of Mathematics, Hanyang University, Seoul, Republic of Korea; 5 Department of Internal Medicine, College of Medicine, Hanyang University Guri Hospital, Guri, Republic of Korea; 6 Department of Statistics and Data Science, College of Commerce and Economics, Yonsei University, Seoul, Republic of Korea; 7 Department of Applied Statistics, College of Commerce and Economics, Yonsei University, Seoul, Republic of Korea; 8 Department of Internal Medicine, School of Medicine, Kyungpook National University, Daegu, Republic of Korea; 9 Department of Internal Medicine, Yonsei University College of Medicine AIDS Research Institute, Yonsei University College of Medicine, Seoul, Republic of Korea; 10 Department of Internal Medicine, Korea University College of Medicine, Seoul, Republic of Korea; 11 Division of Infectious Disease, Department of Internal Medicine, Incheon St. Mary’s Hospital, College of Medicine, The Catholic University of Korea, Seoul, Republic of Korea; 12 Department of Internal Medicine, Yonsei University Wonju College of Medicine, Wonju, Republic of Korea; 13 Division of Infectious Diseases, Department of Internal Medicine, Inha University School of Medicine, Incheon, Republic of Korea; 14 Division of Clinical Research, Center for Emerging Virus Research, National Institute of Infectious Disease, Korea National Institute of Health (KNIH), Cheongwon-gun, Republic of Korea

**Keywords:** Risk factors, Medical research, Epidemiology

## Abstract

The beginning of human immunodeficiency virus (HIV) infection treatment depends on various factors, which are significantly correlated with the initial CD4 cell number. However, a covariate correlation between these factors may not reflect the correct outcome variable. Thus, we evaluated the effects of a combination of fixed factors (reduced dimensions), which determine when to start treatment for the first time, on short-term outcome, long-term outcome, and survival, considering correlations between factors. Multiple correspondence analysis was performed on variables obtained from 925 patients who participated in a Korean HIV/acquired immunodeficiency syndrome cohort study (2006–2017). Five reduced dimension groups were derived according to clinical data, viral load, CD4 cell count at diagnosis, initial antiretroviral therapy, and others. The dimension group with high initial viral loads (55,000 copies/mL) and low CD4 cell counts (< 200 cells/mm^3^) should start treatment promptly after diagnosis. Groups with high initial CD4 cell counts (> 350 cells/mm^3^) that did not require immediate treatment according to previous guidelines had a higher failure rate for long-term relative CD4 recovery. Our results highlight the importance of early diagnosis and treatment to positively influence long-term disease outcomes, even if the initial immune status is poor, given the patient’s combination of early diagnostic symptoms.

## Introduction

In the past, initiation of antiretroviral therapy (ART) was not expedited if the initial immune status was good. However, the treatment guidelines have recently been revised, and treatment initiation is recommended immediately after diagnosis. The clinical course of treatment in human immunodeficiency virus (HIV)-infected individuals and the decision about when to start treatment are related to various factors (such as age and co-infections) that show a significant correlation with the initial number of CD4 cells^1–4^. It is important to determine the optimal timing for treatment to achieve the best outcome over the long and short terms. However, in previous studies, the correlation between these factors (covariates) was not sufficiently considered, and the results were presented in the form of simply correcting the factors in a regression equation^5,6^.

Therefore, it is essential to consider a strong correlation between variables to prevent the over- or under-estimation of the estimated value of outcome variables. In reality, multivariate models and multiple or multivariable models are generally used interchangeably in health science and medicine, but these two models are different^7^. In particular, if the number of variables to be corrected increases, the dimension increases and, consequently, the distance between coordinate values increases, which is not suitable for the multi-model under the linear assumption. In this case, the problem can be resolved through dimensionality reduction, which is a method of multivariate analysis^8–10^. In a typical multivariable model, if the correlation of the main independent variable with the dependent variable can be presented after adjusting for some covariates, the multivariate analysis can identify the combination of factors that exhibits the greatest correlation with the dependent variable^11^. The characteristics of the patient described through the combination of factors are more effective and realistic when explaining the outcome to the actual patient^12^.

This study aimed to evaluate the effects of a combination of fixed factors, which determine when to start treatment for the first time, on the short-term outcomes (endpoint of primary treatment), long-term outcomes (last follow-up), and survival with consideration of the correlation between these fixed factors at initial ART.

## Results

### Derivation of multivariate factor characteristics (reduced dimensions) at the start of treatment

**Figure 1:**
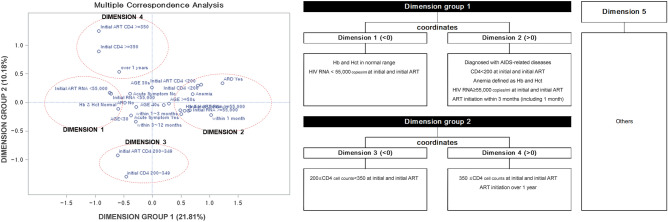
Scatter diagram and characteristics of reduced dimensions by multiple correspondence analysis (MCA) ART, antiretroviral therapy; Hb, hemoglobin; Hct, hematocrit; AIDS, acquired immunodeficiency syndrome.

The variables with a significant effect on the short-term/long-term clinical outcomes were used in the Multiple Correspondence Analysis (MCA, Supplementary Tables 1 and 2). The five reduced dimensions derived from this analysis were: (1) patients with an HIV VL < 55,000 copies/mL at the time of initial treatment with no baseline anemia; (2) patients with a diagnosis of an AIDS-defining disease, CD4 cell count < 200 cells/mm^3^ at diagnosis and initial treatment with baseline anemia, an HIV VL ≥ 55,000 copies/mL at diagnosis, and the start of treatment within 3 months from diagnosis to treatment; (3) patients with a CD4 cell count of 200–350 cells/mm^3^ at diagnosis and initial treatment; (4) patients with a CD4 cell count ≥ 350 cells/mm^3^ initially and at the start of initial treatment with > 1 year from diagnosis to treatment; and (5) patients who did not fall under any of the four aforementioned categories (Fig. 1).

### Effect of reduced dimensions on the short-term disease outcomes

There were 262 patients (28.3%) in reduced dimension 1, 81 (8.8%) in reduced dimension 2, 141 (15.2%) in reduced dimension 3, and 26 (2.8%) in reduced dimension 4. Additionally, patients with four reduced dimensions accounted for 55.1% of the total. Comparison of the results of short-term treatment showed that those patients with a CD4 cell count of 200–350 cells/mm^3^ at diagnosis and initial treatment showed the highest success rate of 63.1%, followed by patients with a VL of < 55,000 copies/mL and no anemia at baseline (59.9%). In contrast, the success rate was only 46.2% in patients who started treatment late (reduced dimension 4) because of a good initial immune status; however, the short-term treatment prognosis was good, as only 1 patient (3.9%) failed short-term treatment. The group with an AIDS-defining disease at the time of diagnosis with a CD4 cell count < 200 cells/mm^3^ and VL ≥ 55,000 copies/mL who started treatment within 1 month after diagnosis because of the presence of baseline anemia showed a short-term success rate of only 42% after treatment; the prognosis was the poorest for this group with a failure rate of 8.6%. A statistically significant difference was observed in short-term disease progression between the five reduced dimensions (*P* < 0.0001) (Table 1).

**Table 1:** Relationship between reduced dimensions and short-term clinical outcomes.

N (%)						
	Initial treatment result					
	Total	Success	Viral suppression only	CD4 recovery only	Failure	*P*-value
N	925 (100.0)	499 (71.6)	151 (16.3)	198 (21.4)	77 (8.3)	
Dimensionality reduction by multiple corresponding analysis						
[1] HIV RNA < 55,000 copies/mL at initial diagnosis and initial ART, without anemia	262 (28.3)	157 (59.9)	55 (21.0)	32 (12.2)	18 (6.9)	< 0.0001
[2] Diagnosed AIDS-defined diseases, CD4 count < 200 cells/mm^3^ and HIV RNA ≥ 55,000 copies/mL at initial diagnosis and initial ART, with anemia, ART initiation within 1 month	81 (8.8)	34 (42.0)	10 (12.4)	30 (37.0)	7 (8.6)	
[3] CD4 count 200–350 cells/mm^3^ at initial diagnosis and initial ART	141 (15.2)	89 (63.1)	18 (12.8)	26 (18.4)	8 (5.7)	
[4] CD4 count ≥ 350 cells/mm^3^ at initial diagnosis and initial ART, and ART initiation more than 1 year after diagnosis	26 (2.8)	12 (46.2)	6 (23.1)	7 (26.9)	1 (3.9)	
[5] Others	415 (44.9)	207 (49.9)	62 (14.9)	103 (24.8)	43 (10.4)	

HIV, human immunodeficiency virus; ART, antiretroviral therapy; AIDS, acquired immunodeficiency syndrome.

### Effect of reduced dimensions on the long-term disease outcomes

All reduced dimensions showed statistically significant immunological recovery (*P* < 0.0001) and viral reduction (*P* < 0.0001). Particularly, in dimension 4, the baseline CD4 cell count and that at the start of treatment were very high, and recovered to > 600 cells/mm^3^ after treatment and reached 776 cells/mm^3^ at the last time point. In contrast, immunity in dimension 2 was severely weakened with an initial CD4 cell count of < 50 cells/mm^3^; with immediate treatment, the cell count recovered to approximately 250 cells/mm^3^ and up to 500 cells/mm^3^ at the last time point. However, the log-substituted initial VL differed by a group of reduced dimensions but decreased to an average of approximately 4 after treatment and about 3.5 at the last time point (Table 2).

**Table 2:** Relationship between reduced dimensions and long-term clinical outcomes.

Reduced dimension	Least squares means ± SE				*P*-value (from initial to latest)
	At initial diagnosis	At initial ART	48 weeks after initial ART	At last time point	
CD4 recovery					
Dimension 1	344.1 ± 10.2	303.2 ± 7.7	492.4 ± 12.7	660.7 ± 16.6	< 0.0001
Dimension 2	43.2 ± 18.3	42.3 ± 13.9	247.4 ± 22.9	491.6 ± 29.8	< 0.0001
Dimension 3	265.1 ± 13.9	261.7 ± 10.5	500.2 ± 17.4	639.0 ± 22.6	< 0.0001
Dimension 4	599.5 ± 32.3	445.4 ± 24.5	641.2 ± 40.4	776.7 ± 52.6	< 0.0001
Others	201.1 ± 8.1	164.7 ± 6.1	362.0 ± 10.1	543.6 ± 13.2	< 0.0001

Reduced dimensions	Least squares means ± SE				*p*-value
	At initial diagnosis	At initial ART	32 weeks after initial ART	At last time point	
Viral suppression					
Dimension 1	9.0 ± 0.12	9.1 ± 0.11	3.8 ± 0.11	3.6 ± 0.10	< 0.0001
Dimension 2	12.7 ± 0.22	12.7 ± 0.20	4.4 ± 0.20	3.5 ± 0.18	< 0.0001
Dimension 3	11.3 ± 0.17	11.3 ± 0.15	4.0 ± 0.16	3.5 ± 0.14	< 0.0001
Dimension 4	10.0 ± 0.39	10.6 ± 0.35	4.5 ± 0.36	4.0 ± 0.32	< 0.0001
Others	11.5 ± 0.10	11.6 ± 0.09	4.2 ± 0.09	3.7 ± 0.08	< 0.0001

SE, standard error; ART, antiretroviral therapy.

### Effect of the reduced dimension at the start of initial treatment on the short-term/long-term clinical outcomes after treatment

#### Short-term CD4 recovery and viral suppression

**Figure 2:**
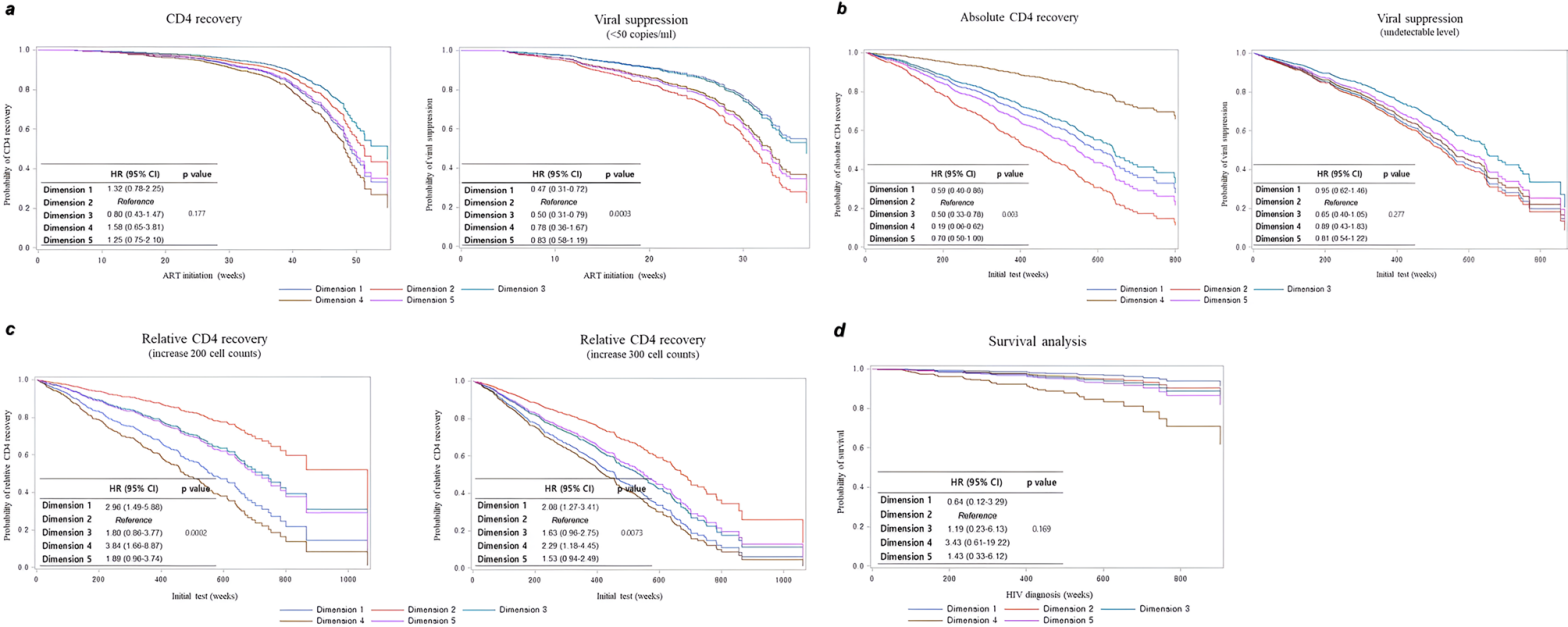
Short- and long-term clinical course of CD4 recovery and viral suppression and hazard ratios of survival by reduced dimension HR, hazard ratio; CI, confidence interval; ART, antiretroviral therapy; HIV, human immunodeficiency virus.

Among the reduced dimensions derived using related factors, dimension 2, which had the lowest treatment success rate, was used as the control; and the effect on short-term CD4 recovery and failure of viral suppression was confirmed in the other groups. No statistically significant difference was observed between the reduced dimensions from the start of treatment throughout the follow-up of about 52 weeks (*P* = 0.177). However, compared with that noted in dimension 2, the hazard ratios (HRs) for CD4 recovery failure in dimensions 1 and 4 were 1.32 (range, 0.78–2.25) and 1.58 (range, 0.65–3.81), respectively, and the risk of failure of recovery was higher but were not statistically significant. Throughout the follow-up of approximately 35 weeks, the risk of not reaching a state of viral suppression was 22% (range, 0.36–1.67) and 53% (range, 0.31–0.72) lower for dimension 4 and 1, respectively, than that for dimension 2. The difference between the groups for viral suppression failure was statistically significant (*P* < 0.0001) (Fig. 2a).

#### Long-term absolute CD4 recovery and viral suppression

When the HRs for the failure of absolute CD4 recovery and undetectable levels of viral suppression were compared from the start of treatment to the last follow-up, the HR for the failure of long-term absolute CD4 recovery was 41% (range, 0.40–0.86), 50% (range, 0.33–0.78), and 81% (range, 0.06–0.62) lower in dimension 1, 3, and 4 than that in dimension 2. All these differences were statistically significant, and the difference between the groups was significant (*P* = 0.003). However, no statistically significant difference was observed between the groups in the reduced dimensions regarding the factors at the start of initial treatment for the failure of viral suppression during the long-term follow-up (*P* = 0.277) (Fig. 2b).

#### Long-term relative CD4 recovery

The criteria for long-term relative CD4 recovery were divided into two categories (an increase of 200 cells/mm^3^ and an increase of 300 cells/mm^3^) for examination. For long-term relative CD4 recovery, there was a statistically significant correlation in both groups (*P* = 0.0002, *P* = 0.0073). Specifically, compared with that noted in the control group (dimension 2), the HRs of CD4 that did not increase by at least 200 cells/mm^3^ at the last time point after treatment compared with the initial CD4 cell count were very high at 2.96 (range, 1.49–5.88) for dimension 1 and 3.84 (range, 1.66–8.87) for dimension 4, both of which were statistically significant. This phenomenon was also observed for the HR of CD4 that did not increase by at least 300 cells/mm^3^ at the last time point. Compared with that noted in dimension 2, the HRs for CD4 recovery failure were significantly higher in dimension 1 (2.08 [range, 1.27–3.41]) and dimension 4 (2.29 [range, 1.18–4.45]) (Fig. 2c).

#### Analysis of survival

In the survival analysis, the HR for death was high in dimension 4 (3.43 [range, 0.61–19.22]) when compared with that in dimension 2, but the difference was not statistically significant. Additionally, no statistically significant difference was found in all five dimensions between the groups (*P* = 0.169) (Fig. 2d).

## Discussion

This study examined the effects of a combination of fixed factors, determining when to start treatment for the first time, on the short-term outcomes, long-term outcomes, and survival in patients with HIV infection. To this end, the dimensions were reduced considering the correlation between the factors, and categorical data were used to facilitate clinical application. The reduced dimensions of each derived factor were independent, and overlapping patients were not included. Other variables except for the five reduced dimensions were not adjusted, as the combination of factors was derived by considering the correlations between the factors at the start of initial treatment that have an effect on short-term and long-term immunological recovery and viral suppression. Patients belonging to dimension 2, which was selected as the control group for MCA, had a high initial HIV VL (above 55,000 copies/mL), initial CD4 cell count at the level of serological AIDS (< 200 cells/mm^3^), diagnosis of an AIDS-defining disease, and anemia; thus, treatment was promptly started after diagnosis (within 3 months). These patients should be administered ART promptly according to treatment guidelines and clinical recommendations. In contrast, patients whose initial CD4 cell count was > 350 cells/mm^3^ did not require immediate treatment, according to past treatment guidelines, and had > 1 year from diagnosis to treatment and a higher failure rate for long-term relative CD4 recovery, although the CD4 cell count at the start of treatment was higher than that in dimension 2. However, most of the reduced dimensions did not show a significant correlation with viral suppression.

When the short- and long-term prognoses were compared with major factors determining the start of treatment and treatment experience, some differences were observed among the factors affecting the short- and long-term outcomes. However, most results were similar to those of previous studies. In the past, the guidelines for starting appropriate treatment were an initial CD4 cell count < 350 cells/mm^3^ and HIV RNA count > 55,000 copies/mL; this criterion was calculated by estimating the point in time when the probability of developing AIDS within 3 years is ≥ 30%^21^. However, the current guideline recommends starting treatment immediately after diagnosis regardless of immune status^22–26^. Among the reduced dimensions (combination of factor components) in this study, the HR for the failure of immunological recovery and viral suppression was high in the long-term prognosis of patients for whom treatment was not recommended as per the guidelines. Ultimately, positive effects on long-term disease outcomes were demonstrated when treatment was started immediately, despite a poor initial immune status, as it leads to relatively good CD4 recovery compared with patients who started treatment late owing to a good initial immune status. This finding is consistent with the results of previous studies, wherein immunological recovery occurred quickly and linearly after treatment initiation regardless of initial CD4 cell counts^27^.

Our results are consistent with previous studies; however, this study, for the first time, proposes a methodology that enables direct explanation to patients in the clinical field in a form easily relatable to their current condition. The characteristics of patients appearing in actual clinical practice can be identified through combinations of each major factor. It is more accurate and practical to use a multivariate model rather than a multiple regression or linear model when using a model with a strong correlation between these factors.

Nevertheless, it cannot be said that the factors affecting disease progression have been sufficiently corrected because the dynamic and fixed factor characteristics at the time of initial treatment were not considered. In addition, the combination of factors derived from this study reflects only the characteristics of Korean individuals infected with HIV who participated in the Korean HIV/AIDS cohort study. Since multivariate analysis, such as dimensionality reduction, comprises a combination of factors derived from the data, the characteristics of the reduced dimensions may vary according to country, race, region, and institution.

The importance of early diagnosis and treatment in HIV prevention and management policies has been sufficiently demonstrated by the results of numerous domestic and foreign studies. Even when infected individuals do not present with the symptoms related to HIV infection or have good immunological status, they should actively participate in treatment at the discretion of the medical staff. If the patient’s initial health condition is relatively poor owing to delayed diagnosis, early treatment will result in immunological recovery, viral suppression, and survival in terms of long-term prognosis that is not significantly different from that of a healthy infected person. Nevertheless, a high CD4 cell count at diagnosis can be retained with a good immune status. As a result, active diagnostic testing is required to detect undiagnosed HIV for its early diagnosis and rapid treatment. Notably, even with a delayed diagnosis, the initiation of treatment should not be delayed. As a result, even if the initial diagnosis of an infected person is fast and the initial immune status is good, the prognosis is poor when treatment is started later after the diagnosis, so prompt treatment is recommended regardless of the infected person’s immune status.

## Methods

### Study design and population

**Figure 3:**
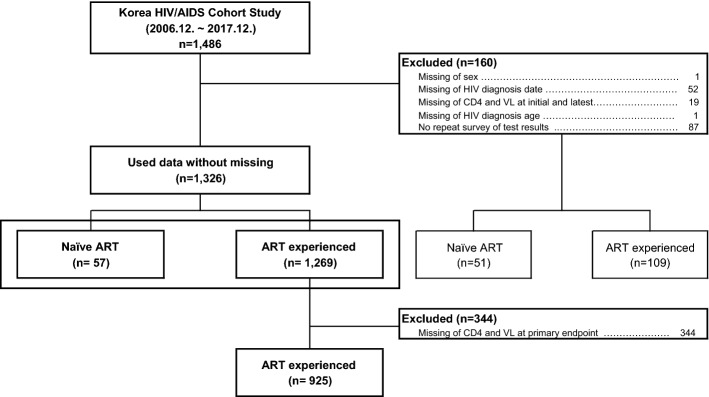
Exclusion criteria of research participants VL, viral load; HIV, human immunodeficiency virus; AIDS, acquired immune deficiency syndrome; ART, antiretroviral therapy;.

The Korea HIV/AIDS Cohort Study was designed as a two-way cohort study that retrospectively examined all medical records from the time of HIV infection diagnosis to enrollment and subsequently conducted a prospective repeat survey every 6 months^13,14^. The data collected from December 2006 to December 2017 were used after refinement^15^. Among the 1,486 patients enrolled in this period, 1269 received ART. Ultimately, 925 patients who had data for all the major variables were included (Fig. 3).

The data used were collected according to the results of an institutional review by the Korea Disease Control and Prevention Agency (Korea HIV/AIDS Cohort Study, 2019-E3201-02). The previously collected secondary data were used, and no additional data were collected for this study nor did invasive damage to the patients occur.

### Ethical approval and informed consent

The Korea HIV/AIDS Cohort study data and resources were collected from adults who were 18 years of age or older at the time of cohort enrollment and who voluntarily agreed to participate. The collection of cohort data was conducted in accordance with the Helsinki Declaration. Furthermore, no additional surveys or samples were collected for the purpose of conducting this study after the cohort data was collected, and the results were derived from analyzing only the data that had already been collected. Because we utilized secondary sources, we were exempt from obtaining patient consent for this study. The study protocol and informed consent waiver were approved by the Institutional Bioethics Committee (approval number HYUIRB 202,008-004), College of Medicine, Hanyang University.

### Study outcomes

The primary endpoints (short-term outcomes) were: (1) CD4 recovery: > 200 cells/mm^3^ within 48 weeks (if the follow-up period was less than 24 weeks, recovery was defined when the CD4 cell count recovered to > 100 cells/mm^3^) from the beginning of ART (the closest test result within 12 weeks prior to the first treatment start date) and (2) viral suppression: 50 copies/mL reached within 32 weeks from the start of initial treatment.

The secondary endpoints (long-term outcomes) were: (1) relative CD4 recovery: when the final CD4 count increased by 200 or ≥ 300 cells/mm^3^ compared with the initial result, (2) absolute CD4 recovery: when the last CD4 count reached ≥ 500 cells/mm^3^ regardless of the initial test result, and (3) viral suppression: when the viral load (VL) was undetectable (20 copies/mL) at the last test result.

### Statistical analysis

The test of suitability for multivariate analysis was defined when the Kaiser Measure of Sampling Adequacy (overall MSA) was ≥ 0.6 and when the *p*-value for correlation between variables (CELLCHI2) on the Butt chart was statistically significant (*P* < 0.05).

Principal component analysis (PCA) is a multivariate analysis method that derives principal components (combinations of factors) by considering the correlation between continuous variables^16^. As most clinical indicators are calculated as continuous variables, PCA may seem suitable, but the actual clinical indicators usually have different normal and abnormal ranges with different concepts of one continuous unit; thus, the results derived using continuous indicators are difficult to apply in clinical practice. Therefore, in research, clinical indicators are usually converted into categorical types. Multiple correspondence analysis (MCA) is a multivariate analysis that derives a combination of factors with consideration of the correlations between categorical data^17–20^. This is a scalable method of PCA that can overcome the limitations of the previous analysis by considering correlations categorically by reducing multiple dimensions. When the correlations between the factors at the start of treatment were considered, the overall MSA was 0.68; the sphericity test was significant (*P* < 0.0001); and the three main components could explain 77.57% of the total (principal component 1, 41.6%; principal component 2, 20.19%; principal component 3, 16.09%), thus, meeting all the basic conditions for multivariate analysis. Hence, it was determined that MCA was required.

This study identified the effect of a combination of changes in short-term and long-term clinical outcomes (CD4 and RNA) by identifying fixed factors at the start of treatment. In order to examine the clinical course over time, using a generalized linear mixed model was used, and the change in the least square means and standard error at each time point was tested for statistical significance. As a quantitative test, international units were converted to copies/mL depending on the type of equipment used to test HIV RNA. Identifying all types of equipment from the surveyed results was impossible. A ‘UNDETECTION’ was recorded separately if the virus was not detected according to the results. Institutions in the cohort used Abbott (1 IU/mL is 0.58 or 0.56 copies/ml depending on the version), Roche (1 IU/mL = 0.5988 copies/ml), and Biomerieux (1 IU/mL = 1 copy/mL) instruments, standardized to the reference standard. The HIV RNA results follow a non-parametric distribution, the results calculated by setting the link function to log-normal under a linear model assumption are presented. Moreover, the effects of the combination of factors on the primary endpoint (the outcomes of initial treatment), secondary endpoint (the last CD4/VL status), and survival were confirmed using Cox hazard regression analysis. All analyses were performed using SAS Enterprise Guide ver. 7.1 (SAS Institute, Cary, NC, USA).

## Supplementary Information

Supplementary Tables.

## Data Availability

The data that support the key findings of this study are available from the Division of Clinical Research, Center for Emerging Virus Research, Korea National Institute of Health, Korea Disease Control and Prevention Agency, but restrictions apply to the availability of these data, which were used under license for only the current study, and so are not publicly available. Data are however available from the authors upon reasonable request and with permission of the Korea Disease Control and Prevention Agency.
