# Supplementary material for: Effect of characteristics on the clinical course at the initiation of treatment for human immunodeficiency virus infection using dimensionality reduction
Source: Sci Rep. 2023 Apr 4;13:5547. doi: 10.1038/s41598-023-31916-x (PMC10073208; doi:10.1038/s41598-023-31916-x)
Supplement: Supplementary file 1 — Supplementary Tables. [file 41598_2023_31916_MOESM1_ESM.docx]

**Appendix Table 1. Factors related to long-term clinical course (changes in CD4 cell count)**

|  | | | **Least Squares Means ± SE** | | | | **p-value (from initial to latest)** | **Initial ART to 48 weeks** | **p-value** | **Initial to latest** | **p-value** | **p-value (Time x factors)** |
| --- | --- | --- | --- | --- | --- | --- | --- | --- | --- | --- | --- | --- |
|  |  |  | **At initial** | **At initial ART** | **48 weeks after initial ART** | **At latest** |  | **Difference ± SE** |  | **Difference ± SE** |  |  |
| **Total** | | | 248.7 ± 6.4 | 215.9 ± 5.0 | 417.8 ± 7.4 | 583.3 ± 9.1 | <0.0001 | 201.9 ± 5.3 | <0.0001 | 344.6 ± 8.5 | <0.0001 |  |
| **Sex** | |  |  |  |  |  |  |  |  |  |  |  |
|  | Men | | 247.1 ± 6.6 | 213.8 ± 5.2 | 417.0 ± 7.6 | 589.9 ± 9.4 | <0.0001 | 203.3 ± 5.5 | <0.0001 | 342.8 ± 8.8 | <0.0001 | 0.527 |
|  | Women | | 271.8 ± 24.5 | 244.5 ± 19.2 | 428.3 ± 28.3 | 640.0 ± 34.8 | <0.0001 | 183.8 ± 20.2 | <0.0001 | 368.3 ± 32.6 | <0.0001 |  |
| **Age at HIV diagnosis (years)** | | |  |  |  |  |  |  |  |  |  |  |
|  | <30 | | 278.5 ± 12.3 | 244.2 ± 9.6 | 461.2 ± 14.1 | 606.5 ± 17.4 | <0.0001 | 217.0 ± 10.1 | <0.0001 | 328.0 ± 16.3 | <0.0001 | 0.006 |
|  | 30-39 | | 250.1 ± 11.9 | 215.9 ± 9.3 | 414.1 ± 13.7 | 601.7 ± 16.9 | <0.0001 | 198.2 ± 9.9 | <0.0001 | 351.6 ± 15.8 | <0.0001 |  |
|  | 40-49 | | 219.8 ± 13.5 | 191.2 ± 10.5 | 398.0 ± 15.5 | 618.4 ± 19.1 | <0.0001 | 206.8 ± 11.1 | <0.0001 | 398.7 ± 17.9 | <0.0001 |  |
|  | ≥50 | | 239.8 ± 13.6 | 206.2 ± 10.6 | 389.6 ± 15.6 | 540.7 ± 19.3 | <0.0001 | 183.4 ± 11.2 | <0.0001 | 300.9 ± 18.0 | <0.0001 |  |
| **Transmission route** | | |  |  |  |  |  |  |  |  |  |  |
|  | Sexual contact | |  |  |  |  |  |  |  |  |  |  |
|  |  | Homo-/bisexual | 260.0 ± 8.1 | 224.3 ± 6.4 | 425.2 ± 9.4 | 603.0 ± 11.6 | <0.0001 | 200.8 ± 6.7 | <0.0001 | 343.0 ± 10.9 | <0.0001 | 0.945 |
|  |  | Heterosexual | 234.5 ± 10.7 | 205.7 ± 8.4 | 411.1 ± 12.3 | 581.4 ± 15.2 | <0.0001 | 205.4 ± 8.8 | <0.0001 | 347.0 ± 14.3 | <0.0001 |  |
|  | Others | | 185.7 ± 37.4 | 162.8 ± 29.3 | 345.6 ± 43.1 | 534.7 ± 53.1 | <0.0001 | 182.8 ± 0.9 | <0.0001 | 349.0 ± 49.9 | <0.0001 |  |
| **Non–AIDS-related disease at diagnosis** | | |  |  |  |  |  |  |  |  |  |  |
|  | Yes | | 264.0 ± 8.3 | 228.5 ± 6.5 | 428.3 ± .5 | 607.3 ± 11.7 | <0.0001 | 199.8 ± 6.8 | <0.0001 | 343.3 ± 11.0 | <0.0001 | 0.758 |
|  | No | | 226.3 ± 10.0 | 197.3 ± 7.9 | 402.4 ± 11.6 | 572.7 ± 14.3 | <0.0001 | 205.0 ± 8.3 | <0.0001 | 346.4 ± 13.4 | <0.0001 |  |
| **AIDS-defined disease at diagnosis** | | |  |  |  |  |  |  |  |  |  |  |
|  | Yes | | 101.1 ± 13.7 | 89.2 ± 10.6 | 288.2 ± 16.3 | 513.9 ± 20.7 | <0.0001 | 199.0 ± 12.1 | <0.0001 | 412.8 ± 19.4 | <0.0001 | 0.0002 |
|  | No | | 283.2 ± 6.6 | 245.4 ± 5.1 | 448.1 ± 7.9 | 611.8 ± 10.0 | <0.0001 | 202.6 ± 5.9 | <0.0001 | 328.7 ± 9.4 | <0.0001 |  |
| **Acute infection symptom** | | |  |  |  |  |  |  |  |  |  |  |
|  | Yes | | 202.4 ± 9.4 | 187.4 ± 7.5 | 403.1 ± 11.1 | 587.6 ± 13.7 | <0.0001 | 215.7 ± 7.9 | <0.0001 | 385.2 ± 12.7 | <0.0001 | <0.0001 |
|  | No | | 285.3 ± 8.4 | 238.4 ± 6.6 | 429.4 ± 9.9 | 597.8 ± 12.1 | <0.0001 | 191.0 ± 7.0 | <0.0001 | 312.5 ± 11.3 | <0.0001 |  |
| **Initial CD4 cell count (cell count/mm^3^)** | | |  |  |  |  |  |  |  |  |  |  |
|  | <200 | | 84.3 ± 4.4 | 94.1 ± 4.8 | 286.5 ± 9.2 | 471.0 ± 12.3 | <0.0001 | 192.4 ± 7.9 | <0.0001 | 386.7 ± 12.3 | <0.0001 | <0.0001 |
|  | 200-349 | | 270.1 ± 5.5 | 262.6 ± 6.0 | 476.2 ± 11.4 | 646.8 ± 15.4 | <0.0001 | 213.6 ± 9.8 | <0.0001 | 376.8 ± 15.4 | <0.0001 |  |
|  | ≥350 | | 506.2 ± 5.8 | 372.6 ± 6.3 | 577.9 ± 12.0 | 743.4 ± 16.1 | <0.0001 | 205.3 ± 10.3 | <0.0001 | 237.2 ± 16.1 | <0.0001 |  |
| **Initial viral load (copies/mL)** | | |  |  |  |  |  |  |  |  |  |  |
|  | <55,000 | | 322.1 ± 8.7 | 271.0 ± 6.9 | 455.3 ± 10.6 | 622.4 ± 13.1 | <0.0001 | 184.4 ± 7.6 | <0.0001 | 300.3 ± 12.2 | <0.0001 | <0.0001 |
|  | ≥55,000 | | 182.7 ± 8.2 | 166.4 ± 6.5 | 384.1 ± 10.0 | 567.2 ± 12.5 | <0.0001 | 217.7 ± 7.2 | <0.0001 | 384.4 ± 11.6 | <0.0001 |  |
| **CD4 cell count at initial ART (cell count/mm^3^)** | | |  |  |  |  |  |  |  |  |  |  |
|  | <200 | | 109.8 ± 6.3 | 87.9 ± 3.2 | 282.0 ± 8.2 | 477.9 ± 11.9 | <0.0001 | 194.1 ± 7.7 | <0.0001 | 368.1 ± 12.4 | <0.0001 | 0.002 |
|  | 200-349 | | 321.4 ± 7.1 | 271.9 ± 3.6 | 479.6 ± 9.3 | 647.2 ± 13.4 | <0.0001 | 207.6 ± 8.7 | <0.0001 | 325.8 ± 14.0 | <0.0001 |  |
|  | ≥350 | | 491.7 ± 10.9 | 465.0 ± 5.6 | 676.9 ± 14.2 | 810.4 ± 20.5 | <0.0001 | 211.9 ± 13.2 | <0.0001 | 318.7 ± 21.3 | <0.0001 |  |
| **Viral load at initial ART (copies/mL)** | | |  |  |  |  |  |  |  |  |  |  |
|  | <55,000 | | 317.5 ± 9.0 | 279.5 ± 6.9 | 463.8 ± 10.8 | 627.2 ± 13.4 | <0.0001 | 184.3 ± 7.8 | <0.0001 | 309.7 ± 2.5 | <0.0001 | 0.001 |
|  | ≥55,000 | | 191.5 ± 8.2 | 162.9 ± 6.3 | 379.6 ± 9.8 | 565.1 ± 12.2 | <0.0001 | 216.6 ± 7.1 | <0.0001 | 373.6 ± 11.4 | <0.0001 |  |
| **Hematological factors** | | |  |  |  |  |  |  |  |  |  |  |
|  | White blood cells | |  |  |  |  |  |  |  |  |  |  |
|  |  | Normal | 286.8 ± 7.4 | 248.8 ± 5.8 | 451.3 ± 8.7 | 631.0 ± 10.8 | <0.0001 | 202.5 ± 6.4 | <0.0001 | 344.2 ± 10.3 | <0.0001 | 0.431 |
|  |  | Leukopenia | 162.9 ± 12.0 | 138.9 ± 9.4 | 334.1 ± 14.1 | 501.7 ± 17.5 | <0.0001 | 195.2 ± 10.4 | <0.0001 | 338.9 ± 16.7 | <0.0001 |  |
|  |  | Leukocytosis | 222.6 ± 35.9 | 184.9 ± 27.9 | 421.4 ± 42.1 | 592.7 ± 52.1 | <0.0001 | 236.6 ± 30.9 | <0.0001 | 370.1 ± 49.9 | <0.0001 |  |
|  |  | Unknown | 162.2 ± 34.1 | 169.7 ± 26.5 | 382.2 ± 39.9 | 537.7 ± 49.4 | <0.0001 | 212.5 ± 29.3 | <0.0001 | 375.6 ± 47.3 | <0.0001 |  |
|  | Hemoglobin and Hematocrit | |  |  |  |  |  |  |  |  |  |  |
|  |  | Normal | 322.3 ± 8.0 | 277.4 ± 6.2 | 481.7 ± 9.6 | 644.6 ± 12.2 | <0.0001 | 204.3 ± 7.2 | <0.0001 | 322.4 ± 11.6 | <0.0001 | 0.006 |
|  |  | Anemia | 164.2 ± 8.9 | 143.6 ± 6.9 | 341.6 ± 10.7 | 534.2 ± 13.6 | <0.0001 | 198.0 ± 8.0 | <0.0001 | 370.0 ± 12.9 | <0.0001 |  |
|  |  | Unknown | 157.2 ± 32.5 | 161.1 ± 25.1 | 375.8 ± 39.0 | 530.5 ± 49.4 | <0.0001 | 214.7 ± 29.3 | <0.0001 | 373.4 ± 47.1 | <0.0001 |  |
|  | Platelets | |  |  |  |  |  |  |  |  |  |  |
|  |  | Normal | 266.6 ± 7.0 | 229.4 ± 5.5 | 429.0 ± 8.2 | 609.9 ± 10.0 | <0.0001 | 199.6 ± 5.9 | <0.0001 | 343.3 ± 9.5 | <0.0001 | 0.157 |
|  |  | Thrombocytopenia | 175.3 ± 15.8 | 157.3 ± 12.4 | 369.3 ± 8.4 | 521.2 ± 22.6 | <0.0001 | 212.0 ± 13.2 | <0.0001 | 345.9 ± 21.4 | <0.0001 |  |
|  |  | Unknown | 158.7 ± 35.5 | 164.0 ± 27.9 | 374.7 ± 41.5 | 529.1 ± 50.9 | <0.0001 | 210.7 ± 29.8 | <0.0001 | 370.4 ± 48.1 | <0.0001 |  |
| **Duration between diagnosis and initial ART** | | |  |  |  |  |  |  |  |  |  |  |
|  | Within 1 month | | 136.2 ± 16.4 | 140.4 ± 13.5 | 392.8 ± 20.4 | 551.3 ± 25.2 | <0.0001 | 252.4 ± 14.6 | <0.0001 | 415.1 ± 23.3 | <0.0001 | <0.0001 |
|  | 1-3 months | | 174.5 ± 11.3 | 179.3 ± 9.2 | 379.1 ± 14.0 | 561.7 ± 17.3 | <0.0001 | 199.8 ± 10.0 | <0.0001 | 387.2 ± 16.0 | <0.0001 |  |
|  | 3-12 months | | 270.3 ± 10.9 | 249.2 ± 8.9 | 458.4 ± 13.5 | 615.1 ± 16.7 | <0.0001 | 209.1 ± 9.7 | <0.0001 | 344.8 ± 15.4 | <0.0001 |  |
|  | Over 1 year | | 342.1 ± 10.7 | 248.5 ± 8.8 | 424.1 ± 13.2 | 618.5 ± 16.4 | <0.0001 | 175.7 ± 9.5 | <0.0001 | 276.3 ± 15.1 | <0.0001 |  |

White blood cells: Normal (4,000-10,000 cells/mm^3^), Leukopenia (<4,000 cells/mm^3^), Leukocytosis (≥10,000 cells/mm^3^); Hemoglobin and Hematocrit: Normal (Hb Men ≥13.5 g/dL, Women ≥12 g/dL or Hct Men ≥40%, Women ≥38%), Anemia (Hb Men <13.5 g/dL, Women <12 g/dL or Hct Men <40%, Women <38%); Platelets: Normal (≥150,000 cells/μL), Thrombocytopenia (<150,000 cells/μL)

**Appendix Table 2. Factors related to long-term clinical course (changes in HIV viral load)**

|  | | | **Least Squares Means ± SE** | | | | **p-value (from initial to latest)** | **Initial ART to 32 weeks** | **p-value** | **Initial to latest** | **p-value** | **p-value (Time x factors)** |
| --- | --- | --- | --- | --- | --- | --- | --- | --- | --- | --- | --- | --- |
|  |  |  | **At initial** | **At initial ART** | **32 weeks after initial ART** | **At latest** |  | **Difference ± SE** |  | **Difference ± SE** |  |  |
| **Total** | | | 10.8 ± 0.08 | 10.9 ± 0.07 | 4.1 ± 0.06 | 3.6 ± 0.05 | <0.0001 | -6.8 ± 0.09 | <0.0001 | -7.2 ± 0.09 | <0.0001 |  |
| **Sex** | |  |  |  |  |  |  |  |  |  |  |  |
|  | Men | | 10.9 ± 0.08 | 10.9 ± 0.07 | 4.1 ± 0.06 | 3.6 ± 0.05 | <0.0001 | -6.8 ± 0.09 | <0.0001 | -7.2 ± 0.09 | <0.0001 | 0.092 |
|  | Women | | 10.5 ± 0.29 | 11.0 ± 0.27 | 4.1 ± 0.23 | 3.5 ± 0.20 | <0.0001 | -6.9 ± 0.33 | <0.0001 | -6.9 ± 0.35 | <0.0001 |  |
| **Age at HIV diagnosis (years)** | | |  |  |  |  |  |  |  |  |  |  |
|  | <30 | | 10.7 ± 0.15 | 10.8 ± 0.14 | 4.3 ± 0.12 | 3.9 ± 0.10 | <0.0001 | -6.5 ± 0.17 | <0.0001 | -6.8 ± 0.17 | <0.0001 | 0.031 |
|  | 30-39 | | 10.7 ± 0.14 | 10.8 ± 0.13 | 4.0 ± 0.11 | 3.7 ± 0.10 | <0.0001 | -6.8 ± 0.16 | <0.0001 | -7.0 ± 0.17 | <0.0001 |  |
|  | 40-49 | | 11.0 ± 0.16 | 11.0 ± 0.15 | 3.9 ± 0.13 | 3.4 ± 0.11 | <0.0001 | -7.1 ± 0.18 | <0.0001 | -7.6 ± 0.19 | <0.0001 |  |
|  | ≥50 | | 11.0 ± 0.16 | 11.1 ± 0.15 | 4.1 ± 0.13 | 3.4 ± 0.11 | <0.0001 | -6.9 ± 0.18 | <0.0001 | -7.6 ± 0.19 | <0.0001 |  |
| **Transmission route** | | |  |  |  |  |  |  |  |  |  |  |
|  | Sexual contact | |  |  |  |  |  |  |  |  |  | 0.325 |
|  |  | Homo-/bisexual | 10.7 ± 0.10 | 10.8 ± 0.09 | 4.1 ± 0.08 | 3.6 ± 0.07 | <0.0001 | -6.6 ± 0.11 | <0.0001 | -7.1 ± 0.12 | <0.0001 |  |
|  |  | Heterosexual | 11.1 ± 0.13 | 11.2 ± 0.12 | 4.1 ± 0.10 | 3.7 ± 0.09 | <0.0001 | -7.1 ± 0.14 | <0.0001 | -7.4 ± 0.15 | <0.0001 |  |
|  | Others | | 10.9 ± 0.45 | 11.0 ± 0.42 | 3.7 ± 0.36 | 3.5 ± 0.31 | <0.0001 | -7.3 ± 0.51 | <0.0001 | -7.4 ± 0.53 | <0.0001 |  |
| **Non–AIDS-related disease diagnosis** | | |  |  |  |  |  |  |  |  |  |  |
|  | Yes | | 10.9 ± 0.10 | 10.9 ± 0.09 | 4.1 ± 0.08 | 3.6 ± 0.07 | <0.0001 | -6.8 ± 0.11 | <0.0001 | -7.4 ± 0.12 | <0.0001 | 0.057 |
|  | No | | 10.7 ± 0.12 | 10.9 ± 0.11 | 4.1 ± 0.10 | 3.7 ± 0.08 | <0.0001 | -6.8 ± 0.14 | <0.0001 | -7.0 ± 0.14 | <0.0001 |  |
| **AIDS-defined disease diagnosis** | | |  |  |  |  |  |  |  |  |  |  |
|  | Yes | | 11.8 ± 0.17 | 11.9 ± 0.16 | 4.3 ± 0.14 | 3.6 ± 0.12 | <0.0001 | -7.6 ± 0.20 | <0.0001 | -8.2 ± 0.21 | <0.0001 | <0.0001 |
|  | No | | 10.6 ± 0.08 | 10.7 ± 0.08 | 4.1 ± 0.07 | 3.6 ± 0.06 | <0.0001 | -6.6 ± 0.10 | <0.0001 | -7.0 ± 0.10 | <0.0001 |  |
| **Acute infection symptom** | | |  |  |  |  |  |  |  |  |  |  |
|  | Yes | | 11.7 ± 0.11 | 11.6 ± 0.10 | 4.1 ± 0.09 | 3.6 ± 0.08 | <0.0001 | -7.5 ± 0.13 | <0.0001 | -8.1 ± 0.13 | <0.0001 | <0.0001 |
|  | No | | 10.2 ± 0.10 | 10.4 ± 0.09 | 4.1 ± 0.08 | 3.7 ± 0.07 | <0.0001 | -6.3 ± 0.11 | <0.0001 | -6.5 ± 0.12 | <0.0001 |  |
| **Initial CD4 cell count (cell count/mm^3^)** | | |  |  |  |  |  |  |  |  |  |  |
|  | <200 | | 11.7 ± 0.11 | 11.7 ± 0.10 | 4.3 ± 0.09 | 3.7 ± 0.08 | <0.0001 | -7.4 ± 0.13 | <0.0001 | -7.9 ± 0.13 | <0.0001 | <0.0001 |
|  | 200-349 | | 10.5 ± 0.13 | 10.5 ± 0.13 | 4.1 ± 0.11 | 3.5 ± 0.10 | <0.0001 | -6.4 ± 0.16 | <0.0001 | -7.0 ± 0.16 | <0.0001 |  |
|  | ≥350 | | 9.8 ± 0.14 | 10.1 ± 0.13 | 3.8 ± 0.12 | 3.5 ± 0.10 | <0.0001 | -6.3 ± 0.17 | <0.0001 | -6.3 ± 0.17 | <0.0001 |  |
| **Initial viral load (copies/mL)** | | |  |  |  |  |  |  |  |  |  |  |
|  | <55,000 | | 9.0 ± 0.07 | 9.5 ± 0.08 | 3.9 ± 0.09 | 3.6 ± 0.08 | <0.0001 | -5.6 ± 0.11 | <0.0001 | -5.4 ± 0.10 | <0.0001 | <0.0001 |
|  | ≥55,000 | | 12.5 ± 0.07 | 12.2 ± 0.08 | 4.3 ± 0.08 | 3.6 ± 0.07 | <0.0001 | -7.9 ± 0.11 | <0.0001 | -8.8 ± 0.10 | <0.0001 |  |
| **CD4 cell count at initial ART (cell count/mm^3^)** | | |  |  |  |  |  |  |  |  |  |  |
|  | <200 | | 11.6 ± 0.11 | 11.6 ± 0.10 | 4.3 ± 0.09 | 3.7 ± 0.08 | <0.0001 | -7.4 ± 0.12 | <0.0001 | -7.9 ± 0.13 | <0.0001 | <0.0001 |
|  | 200-349 | | 10.3 ± 0.12 | 10.4 ± 0.11 | 4.0 ± 0.10 | 3.5 ± 0.09 | <0.0001 | -6.4 ± 0.14 | <0.0001 | -6.7 ± 0.15 | <0.0001 |  |
|  | ≥350 | | 9.9 ± 0.18 | 9.9 ± 0.17 | 3.8 ± 0.15 | 3.6 ± 0.13 | <0.0001 | -6.1 ± 0.21 | <0.0001 | -6.4 ± 0.22 | <0.0001 |  |
| **Viral load at initial ART (copies/mL)** | | |  |  |  |  |  |  |  |  |  |  |
|  | <55,000 | | 9.3 ± 0.09 | 9.1 ± 0.07 | 3.9 ± 0.09 | 3.6 ± 0.08 | <0.0001 | -5.3 ± 0.11 | <0.0001 | -5.8 ± 0.12 | <0.0001 | <0.0001 |
|  | ≥55,000 | | 12.1 ± 0.08 | 12.4 ± 0.06 | 4.3 ± 0.08 | 3.7 ± 0.07 | <0.0001 | -8.1 ± 0.10 | <0.0001 | -8.4 ± 0.11 | <0.0001 |  |
| **Hematological factors** | | |  |  |  |  |  |  |  |  |  |  |
|  | White blood cells | |  |  |  |  |  |  |  |  |  |  |
|  |  | Normal | 10.6 ± 0.09 | 10.7 ± 0.09 | 4.0 ± 0.07 | 3.6 ± 0.06 | <0.0001 | -6.6 ± 0.10 | <0.0001 | -7.0 ± 0.11 | <0.0001 | 0.017 |
|  |  | Leukopenia | 11.3 ± 0.15 | 11.4 ± 0.14 | 3.4 ± 0.12 | 3.8 ± 0.10 | <0.0001 | -7.0 ± 0.17 | <0.0001 | -7.5 ± 0.18 | <0.0001 |  |
|  |  | Leukocytosis | 10.7 ± 0.44 | 11.5 ± 0.41 | 3.7 ± 0.36 | 3.3 ± 0.31 | <0.0001 | -7.7 ± 0.51 | <0.0001 | -7.4 ± 0.53 | <0.0001 |  |
|  |  | Unknown | 11.3 ± 0.42 | 11.4 ± 0.39 | 3.5 ± 0.34 | 3.4 ± 0.29 | <0.0001 | -7.9 ± 0.48 | <0.0001 | -7.9 ± 0.50 | <0.0001 |  |
|  | Hemoglobin and Hematocrit | |  |  |  |  |  |  |  |  |  |  |
|  |  | Normal | 10.3 ± 0.10 | 10.3 ± 0.09 | 3.9 ± 0.08 | 3.6 ± 0.07 | <0.0001 | -6.4 ± 0.12 | <0.0001 | -6.8 ± 0.12 | <0.0001 | <0.0001 |
|  |  | Anemia | 11.4 ± 0.11 | 11.6 ± 0.10 | 4.3 ± 0.09 | 3.7 ± 0.08 | <0.0001 | -7.2 ± 0.13 | <0.0001 | -7.7 ± 0.14 | <0.0001 |  |
|  |  | Unknown | 11.6 ± 0.41 | 11.6 ± 0.38 | 3.6 ± 0.34 | 3.4 ± 0.29 | <0.0001 | -8.0 ± 0.47 | <0.0001 | -8.2 ± 0.50 | <0.0001 |  |
|  | Platelets | |  |  |  |  |  |  |  |  |  |  |
|  |  | Normal | 10.7 ± 0.08 | 10.8 ± 0.08 | 4.1 ± 0.07 | 3.6 ± 0.06 | <0.0001 | -6.7 ± 0.10 | <0.0001 | -7.1 ± 0.10 | <0.0001 | 0.188 |
|  |  | Thrombocytopenia | 11.3 ± 0.19 | 11.5 ± 0.18 | 4.5 ± 0.15 | 3.9 ± 0.13 | <0.0001 | -7.0 ± 0.22 | <0.0001 | -7.4 ± 0.23 | <0.0001 |  |
|  |  | Unknown | 11.5 ± 0.43 | 11.5 ± 0.40 | 3.5 ± 0.34 | 3.4 ± 0.30 | <0.0001 | -8.0 ± 0.49 | <0.0001 | -8.1 ± 0.51 | <0.0001 |  |
| **Duration between diagnosis and initial ART** | | |  |  |  |  |  |  |  |  |  |  |
|  | Within 1 month | | 12.3 ± 0.20 | 12.4 ± 0.19 | 4.2 ± 0.17 | 3.6 ± 0.15 | <0.0001 | -8.2 ± 0.23 | <0.0001 | -8.8 ± 0.24 | <0.0001 | <0.0001 |
|  | 1-3 months | | 11.3 ± 0.14 | 11.3 ± 0.13 | 3.9 ± 0.12 | 3.4 ± 0.10 | <0.0001 | -7.4 ± 0.16 | <0.0001 | -7.9 ± 0.17 | <0.0001 |  |
|  | 3-12 months | | 10.7 ± 0.13 | 10.7 ± 0.12 | 4.2 ± 0.11 | 3.7 ± 0.10 | <0.0001 | -6.5 ± 0.15 | <0.0001 | -7.0 ± 0.16 | <0.0001 |  |
|  | Over 1 years | | 10.0 ± 0.13 | 10.2 ± 0.12 | 4.2 ± 0.11 | 3.8 ± 0.10 | <0.0001 | -6.0 ± 0.15 | <0.0001 | -6.1 ± 0.16 | <0.0001 |  |

Distribution defined as log-normal, log scale translation; 1.25 as 20, 4 as 10,000, 6 as 1,000,000, 6.5 as 3,000,000, 7 as 10,000,000, 10 as 10,000,000,000

White blood cells: Normal (4,000-10,000 cells/mm^3^), Leukopenia (<4,000 cells/mm^3^), Leukocytosis (≥10,000 cells/mm^3^); Hemoglobin and Hematocrit: Normal (Hb Men ≥13.5 g/dL, Women ≥12 g/dL or Hct Men ≥40%, Women ≥38%), Anemia (Hb Men <13.5 g/dL, Women <12 g/dL or Hct Men <40%, Women <38%); Platelets: Normal (≥150,000 cells/μL), Thrombocytopenia (<150,000 cells/μL)
